# Overview of task shifting guidelines in Japan: from radiologists to radiological technologists

**DOI:** 10.1007/s11604-025-01774-w

**Published:** 2025-05-05

**Authors:** Aki Kido, Kazuko Ohno, Kei Yamada, Koichiro Yamakado, Takao Hiraki, Takashi Mizowaki, Noriko Aida, Noriko Oyama-Manabe, Naoki Kodama, Katsuhiko Ueda, Shigeki Aoki, Noriyuki Tomiyama

**Affiliations:** 1 Department of Radiology, Toyama University Hospital, 2630 Sugitani, Toyama City, 930-0152 Japan; 2 Department of Radiological Technology, Kyoto University of Medial Science, 1-3 Koyamahigashiimakita, Sonobe-cho, Nantan City, Kyoto, 622-0041 Japan; 3 Department of Radiology, Kyoto Prefectural University of Medicine, Kajii-cho, Kawaramachi-Hirokoji, Kamigyo-ku, Kyoto, 602-8566 Japan; 4 Department of Radiology, The Hospital of Hyogo College of Medicine, 1-1 Mukogawa-Cho, Nishinomiya City, Hyogo 663-8501 Japan; 5 Department of Radiology, Okayama University, 2-5-1 Shikata-cho, Kita-ku, Okayama City, 700-8558 Japan; 6 Department of Radiation Oncology and Image-Applied Therapy, Graduate School of Medicine, Kyoto University, 54 Shogoinkawahara-cho, Sakyo-ku, Kyoto City, Kyoto, 606-8507 Japan; 7 Department of Diagnostic Radiology, Yokohama City University Graduate School of Medicine, 3-9 Fukuura, Kanazawa-ku, Yokohama City, Kanagawa 236-0004 Japan; 8 Department of Radiology, Jichi Medical University Saitama Medical Center, 1-847 Amanuma-cho, Ohmiya-ku, Saitama, 330-0834 Japan; 9 Department of Radiological Technology, Faculty of Medical Technology, Niigata University of Health and Welfare, 1398 Shimami-cho, Kita-ku, Niigata City, 950-3198 Japan; 10 Department of Radiological Sciences, School of Health Sciences at Narita, International University of Health and Welfare, 4-3, Kozunomori, Narita-shi, Chiba-ken 286-8686 Japan; 11 Health Data Science, Department of Radiology/Data Science, Graduate School of Medicine, Juntendo University, 6-8-1 Hinode, Urayasu, Chiba 279-0013 Japan; 12 Department of Radiology, Osaka University Graduate School of Medicine, 2-15 Yamadaoka, Suita City, Osaka 565-0871 Japan

**Keywords:** Task shifting and sharing, Radiological technologists, Guideline, IGRT, STAT

## Abstract

As one of the key pillars of work style reform for physicians, task shifting and sharing from radiologists to radiological technologists has been considered. In May 2021, the Radiological Technologists Act was amended, allowing for the expansion of several duties. Alongside these legal and regulatory changes, a notice from Ministry of Health, Labour and Welfare was issued, highlighting tasks to be particularly promoted under the current system prior to the amendment of the Radiological Technologists Act. These amendments authorize radiological technologists to perform advanced and specialized tasks, such as securing venous access for contrast agent administration, which require significantly higher skill levels than their traditional roles. However, the amended legislation did not include specific guidelines, rules, or considerations for the practical implementation of these new duties in daily medical practice, especially from the perspectives of patient safety and quality of care. To address this, the Japan Radiological Society, the Japanese College of Radiology, and the Japan Association of Radiological Technologists collaborated with other related societies to develop guidelines on five key topics:-Guidelines for Safe Conduct of CT/MRI Contrast-Enhanced Examinations: Considering the expanded scope of practice for radiological technologists. -Guidelines for Safe Conduct of Nuclear Medicine Examinations: Aligned with the expanded responsibilities of radiological technologists. -Guidelines for Clinical application of Image-Guided Radiation Therapy (IGRT). -Guidelines for Safe Conduct of Angiography and Interventional Radiology (IR): Adapted for the expanded roles of radiological technologists. -Guidelines for Reporting Findings of STAT Imaging: Addressing urgent conditions with potential impact on life prognosis.

## Introduction

Radiology departments, more than other specialties, require close collaboration among radiological technologists, nurses, and administrative staff. In particular, seamless coordination with radiological technologists is essential to ensure that radiological imaging and treatment procedures are performed safely and maintain consistent quality.

The significant shortage of radiologists in Japan has long been a well-recognized issue [1, 2]. Even data from approximately a decade ago estimated that, relative to the number of CT and MRI examinations, the country required more than twice the number of diagnostic radiologists and at least 1.2 times the number of radiation oncologists [1]. Consequently, Japan has a far greater potential workload for radiologists compared to other countries [2]. Furthermore, the widening regional disparities in radiology services have become a major concern in recent years [2].

In recent years, the increasing complexity and sophistication of medical care, coupled with a growing workload and the need to prioritize patient safety, have significantly increased the burden on physicians. Additionally, workstyle reform measures have imposed restrictions on physicians’ working hours [3]. Against this backdrop, task shifting and sharing from radiologists to radiological technologists have been explored [4].

Compared to United States of America, Japan has been slower to implement task shifting in radiology. For example, in some hospitals, radiologists still perform intravenous access and contrast administration themselves. On the other hand, within specialized areas of radiology, such as nuclear medicine, radiation therapy, and interventional radiology (IR), highly skilled technologists and nurses have emerged. Although related academic societies have recognized such professionals through certifications (e.g., Japan Authorize Organization for Magnetic Resonance Technological Specialist (JMRTS), Board Certified Nuclear Medicine Technologist (BCNMT), Intervention Nursing Expert (IEN), Japan Professional Association Board for Radiotherapy Technologists, and the Japan Professional Accreditation Board of Radiological Technologist for Angiography and Intervention), specific procedures that require these certifications are not clearly defined. In contrast, since 2003, the United States has granted the designation of Radiologist Assistant (RA) to radiological technologists who possess certain education, experience, and qualifications, as certified by the American Registry of Radiological Technologists (ARRT). These RAs are authorized to perform invasive procedures such as pleural effusion aspiration and central venous catheter placement under the supervision of radiologists.

Under this circumstance, Ministry of Health, Labour and Welfare (MHLW) Japan identified task shifting and sharing from radiologists to radiological technologists as a key pillar of workstyle reform for physicians. In May 2021, the Radiology Technicians Act was amended to allow the expansion of several duties for radiological technologists. Alongside these legislative and regulatory amendments, a notice from MHLW was issued, highlighting tasks that could be particularly promoted under the current system, even prior to the law's revision [4]. With these changes, radiological technologists were authorized to perform advanced and specialized tasks, such as securing intravenous access for contrast agent administration—a task that requires significantly higher technical skills than their previous roles. However, the amended legislation did not include specific guidelines, rules, or considerations for the practical implementation of these new duties in daily practice, especially concerning patient safety and the quality of care.

To address this, the Japan Radiological Society (JRS), the Japanese College of Radiology (JCR), and the Japan Association of Radiological Technologists (JART) collaborated with various related societies to develop five comprehensive guidelines. These guidelines aim to ensure the safe and effective implementation of the expanded roles for radiological technologists in clinical practice. This paper introduces the content of these guidelines.

The organizations involved in developing the guidelines are as follows: Japanese Society for Radiation Oncology, Japanese Society of Interventional Radiology, Japanese Society of Nuclear Medicine, Japanese Society of Nuclear Medicine Technology, Japanese Society of Radiological Technology, Japan Professional Accreditation Board of Radiological Technologist for Angiography and Intervention, Japan Society of Medical Physics, and Japanese college for Medical Physics.

## Background leading to legal amendments

In Japan, since around the year of 2000, the increasing sophistication and complexity of medical care, coupled with growing workloads, have highlighted exhaustion in medical settings, raising concerns about the condition of healthcare delivery. In response, the MHLW spearheaded the promotion of "team-based medical care” [5]. This model emphasized collaboration among various medical professionals, leveraging their specialized expertise to provide coordinated, patient-centered care [5]. The targeted professionals included radiological technologists, pharmacists, rehabilitation specialists, occupational therapists, and others. At that time, MHLW provided a framework for tasks that non-physician medical staff could perform. However, for radiological technologists, the scope was limited to tasks such as explaining procedures to patients, with no detailed procedural items specified, and no legal amendments were enacted [6].

In 2017, MHLW initiated a working group to address workstyle reform for physicians. After 22 time of meetings, the group issued a report emphasizing the urgent need to promote task shifting and sharing [6]. This initiative was driven by the government's broader workstyle reform agenda, which included implementing limits on physicians’ overtime work starting in 2024. Beginning in April 2024, the maximum allowable annual overtime for employed physicians engaged in medical practice, excluding exceptional cases, was capped at 960 h [3]. Subsequently, MHLW advanced discussions based on hearings from relevant medical professions. This led to the identification and promotion of tasks that could be shifted or shared from physicians to other healthcare professionals without requiring legal amendments. Furthermore, in 2021, the Radiology Technicians Act was amended, allowing for the expansion of several duties for radiological technologists.

A key stipulation was that radiological technologists must complete designated training programs, implemented by the Japan Association of Radiological Technologists (JART), to perform the newly authorized tasks. These programs, referred to as "Training Program recognised by the Ministry of Health," commenced nationwide at the end of 2021. As of October 2024, 25,517 individuals had completed the practical training, and the number of radiological technologists whose licenses had been updated accordingly continues to grow. The initiative remains ongoing.

## Intravenous access for contrast-enhanced CT and MRI

CT examinations are indispensable in daily clinical practice for assessing patient conditions, from initial diagnosis to preoperative and postoperative evaluations, as well as following after treatment. Because non-contrast CT provides limited information, contrast-enhanced CT and MRI are crucial for enhancing lesion detectability, diagnostic accuracy, vascular anatomy visualization, and blood flow assessment.

Before the legal amendments in Japan, the role of radiological technologists in contrast-enhanced CT and MRI examinations was limited to pressing the start button of contrast media injector and removing the needle after administration. Radiological technologists were not permitted to get intravenous access for contrast media. In addition, in a survey conducted in 2018 targeting JRS members, the most frequently requested task shift pertained to the administration of contrast agents [8]. Consequently, in hospitals where nurses were unavailable in the examination room, physicians often get the intravenous access procedures themselves.

Following the recent amendment, radiological technologists are now permitted, under direct instructions from a physician, to perform the following tasks during contrast-enhanced imaging examinations as part of their auxiliary role in medical practice:

- Securing intravenous access for the contrast injector,
- Connecting the contrast injector to the venous access line,
- Operating the contrast injector for contrast administration, and
- Removing the needle and performing hemostasis after administration [4, 7].

This change enables radiological technologists to independently manage the entire process for contrast-enhanced CT and MRI examinations, from securing intravenous access to conducting imaging and post-imaging needle removal.

When securing intravenous access for contrast injection, radiological technologists must adhere to instructions from a physician regarding the type of contrast agent, injection rate, and other parameters. Unlike standard peripheral intravenous placement, venous access for contrast injection requires securing veins capable of withstanding high-pressure injections and ensuring sufficient diameter. Moreover, as adverse reactions, such as anaphylactic shock, may occur immediately after administration, it is essential to establish a system where physicians can respond appropriately. Thus, a well-prepared environment that prioritizes patient safety is critical.

The decision on who should perform venous access for CT/MRI examinations will be made collaboratively among staff, considering each hospital's specific circumstances. This includes factors such as the number of examinations, location of examination room, as well as the number and roles of radiologists, radiological technologists, and nurses. Some hospitals may choose not to expand the scope of practice for radiological technologists and continue with existing workflows. Additionally, the decision to allow radiological technologists to perform venous access should consider the individual capabilities and skills of each technologist. Hospitals are recommended to establish clear standards and protocols for venous access procedures if such tasks are assigned to radiological technologists.

Of notes, radiological technologists are currently prohibited from performing the following tasks: Blood sampling, Administration of drugs other than iodine-based contrast agents (except saline), Venous access for purposes other than contrast imaging, Puncturing CV ports with specialized needles, and Puncturing central veins or other non-peripheral sites.

## Nuclear medicine

In addition to securing venous access for contrast-enhanced CT/MRI examinations, venous access for nuclear medicine procedures is now permitted. Unlike CT/MRI contrast agents, nuclear medicine involves administering radiopharmaceuticals containing radioactive isotopes, allowing internal radiation exposure to the patient. Through the revision of the Radiological Technologist Act, the administration of radioactive isotopes and their compounds, which had previously been prohibited for radiological technologists, has now been authorized. Nuclear medicine procedures may also include administering sodium iodide capsules orally or having patients inhale gaseous radiopharmaceuticals. With the recent legal amendments, radiological technologists can now manage the entire workflow for nuclear medicine examinations, from securing venous access and administering radiopharmaceuticals to imaging and post-imaging needle removal.

It is important to note, however, that the administration of radiopharmaceuticals is restricted to diagnostic purposes only. Administration for therapeutic purposes, such as radionuclide therapy, is not permitted. Additionally, the administration of pharmacological agents used in stress tests, such as those for cardiac function evaluation, is also prohibited. The following tasks remain beyond the scope of radiological technologists in nuclear medicine: Blood collection (including specimen sampling and glucose measurement), Administration of non-radiopharmaceutical agents (e.g., stress-inducing drugs for testing), Venous access for purposes other than nuclear medicine procedures, Administration of radiopharmaceuticals for radionuclide therapy, and Subcutaneous or intradermal injection of radiopharmaceuticals.

The final verification of whether the prepared radiopharmaceuticals are suitable for the procedure and whether the administered radioactive dose is appropriate remains the responsibility of the nuclear medicine physician or referring physician. When radiological technologists administer radiopharmaceuticals, they must follow specific instructions regarding the selection of the radiopharmaceutical, prescribed radioactivity dose, and actual administered dose. Even when specific radiopharmaceuticals and doses are predetermined for each examination type, physician oversight and instructions are required.

In the examination of nuclear medicine, the accuracy of the administered dose is critical. Both excessive and insufficient doses compromise the quality of the diagnostic procedure, with excessive doses unnecessarily increasing patient radiation exposure. This highlights the importance of proper education and collaboration within the radiology team, with particular attention to radiation exposure management in nuclear medicine.

To mitigate risks of misadministration or incidents, institutions should establish a collaborative framework with their medical safety management offices, ensuring robust protocols for patient safety and error prevention.

## Image-guided radiation therapy (IGRT)

Image-guided Radiation Therapy (IGRT) is a technique designed to reproduce the planned radiation target position as accurately as possible during the course treatment. The definition of IGRT encompasses alignment technology that measures and corrects three-dimensional (3D) positional displacements of patients and/or targets based on two or more 2D images, 3D images, or 3D patient surface information, ensuring that the radiation target position defined by treatment planning is reproduced with high precision [9]. IGRT relies on the alignment of the coordinate centers of the radiation delivery device and the image-guidance system (i.e., the irradiation coordinate center and the alignment coordinate center). This necessitates rigorous Quality Assurance (QA) and Quality Control (QC) procedures for both the radiation delivery and the image-guidance system. Any inaccuracies in QA or QC may result in misaligned targeting, potentially reducing treatment efficacy or increasing the risk of adverse events.

With the promotion of task shifting and task sharing initiatives, it has been proposed that the task of image registration for each irradiation session be transferred from physicians to radiological technologists [4]. In this framework, radiological technologists, under the specific directives of a physician, may perform initial image registration to confirm the accuracy of the irradiation position using the alignment images, after which they can proceed with the radiation delivery. However, if the positional deviation exceeds the acceptable range, the radiological technologist must promptly report the issue to the physician. The decision to continue or halt radiation treatment remains the physician’s responsibility. Radiological technologists are also required to record and manage the results of the image registration process, as outlined in the official notification [4]. In addition, physicians need to check the results of IGRT carried out by radiological technologists during regular check-ups of patients undergoing radiotherapy [9].

“Specific instructions” refer to directives detailed enough to eliminate the need for discretion by the radiological technologist. Each patient undergoing IGRT must have explicit instructions that include, at a minimum, the modality used for image registration, the target for alignment, the permissible tolerance for positional deviation, and the correction axis (e.g., 3-axis or 6-axis correction).

### *Requirements for task shifting/sharing in IGRT*

Physicians must provide radiological technologists with the necessary patient information and detailed instructions to ensure the safe delegation of initial image registration tasks. Radiological technologists must acquire the requisite knowledge and skills for accurate alignment, which can be achieved through participation in multidisciplinary conferences and dedicated education and training programs. Educational and training initiatives should encompass not only theoretical knowledge but also hands-on training, such as on-the-job training (OJT). Additionally, institutions must establish alignment manuals tailored to their operational policies to enhance patient safety and maintain high standards in IGRT practices.

By adopting these measures, hospitals can ensure the safe and effective implementation of task shifting and task sharing in IGRT, maintaining high treatment accuracy and minimizing risks to patients.

## Expanded roles of radiological technologists in IR following legal revisions for task shifting

The area of Interventional Radiology (IR) has seen the greatest expansion in tasks radiological technologists are authorized to perform, due to their newly permitted role in sterile procedures. According to official notification [4], radiological technologists may now engage in the following non-medical acts as part of their support role for interventional radiologists:

- Preparing catheters, guidewires, and related tools to be ready for use.
- Handing these tools to the interventional radiologist.
- Holding catheters and guidewires during procedures.
- Safely storing catheters and guidewires removed by the interventional radiologist in sterile trays.

These tasks, which do not constitute medical acts, should be performed by radiological technologists after receiving sufficient training and guidance from radiologists and nurses regarding entry procedures into sterile areas.

While the scope of tasks has broadened, it remains critical to emphasize that radiological technologists are prohibited from engaging in medical acts, such as manipulating catheters or guidewires. Radiological technologists are not considered secondary operators in IR procedures and must adhere strictly to their supportive role.

### *Overview of expanded tasks in IR*

Given the wide range of newly authorized tasks, the following summarizes the approved procedures for radiological technologists during IR:

### *Pre-procedure*

Before the IR, the attending physician explains to the patient the purpose of the procedure, the specific techniques to be used, potential risks, side effects of contrast agents or medications, and the effects of radiation exposure. With task shifting initiatives, radiological technologists, under the direction of the radiologist, are now permitted to provide standardized explanations to patients regarding angiographic techniques and radiation exposure, and review patient information such as blood test results, medication status, and risk factors in accordance with institutional checklists and report findings to the radiologist. To fulfill these responsibilities effectively, radiological technologists should participate in pre-procedure conferences to share patient information and communicate thoroughly with the radiologist. Additionally, they should be able to provide clear explanations about standardized techniques, radiation doses, and potential side effects of contrast agents.

### *During angiography and IR procedures*

The following tasks have been newly permitted for radiological technologists during angiography and interventional radiology (IR):

-Maintaining sterility for entry into clean zones: Proper handwashing. Wearing sterile gowns and gloves to ensure sterility.
- Preparation of angiography kits: Assembling and organizing angiography kits required for examinations and treatments under sterile conditions.
- Preparing disinfectants such as povidone-iodine and required medications for use.
- Preparation of catheters and guidewires: Preparing catheters, guidewires, and other instruments for use.
- Preparing additional devices, such as inflators for balloon catheters, required during treatment.
- Deployment of sterile angiography drapes: Setting up sterile drapes for the procedure.
- Preparation of automatic injection devices: Connecting specialized syringes and pressure-resistant extension tubes. Removing air from syringes and tubing to prepare for use.
- Preparation of ultrasound devices for puncture: Attaching sterile covers to the probe for confirming puncture sites.
- Preparation and support for specialized devices: Setting up equipment such as IVUS (intravascular ultrasound), OCT (optical coherence tomography), and FFR (fractional flow reserve) systems. Holding instruments to assist the interventional radiologist in their operation.
- Handing over instruments: Passing prepared catheters, guidewires, and other instruments to the interventional radiologist.
- Holding instruments during procedures: Assisting the interventional radiologist by holding catheters and guidewires to facilitate their manipulation.
- Post-procedure instrument management: Safely placing catheters and guidewires removed by the interventional radiologist into sterile trays and cleaning up.

### *Post-procedural patient monitoring*

After angiography or IR, radiological technologists may estimate patients' entrance skin doses. If the maximum skin dose exceeds the institution’s management thresholds or guidelines established by professional societies (e.g., thresholds for deterministic effects), the following actions can be performed under the radiologist’s direction: Providing explanations to patients about radiation exposure and conducting follow-up monitoring for adverse effects.

To successfully implement task shifting/sharing for radiological technologists, collaboration among the multidisciplinary team—including radiologists, nurses, clinical engineers, and clinical laboratory technicians—is essential. These professionals work together within the scope of radiological technologists’ legal framework to ensure safety and proper delegation of non-medical support tasks.

As for the educational and institutional requirements, radiological technologists are required to undergo education and hands-on training on sterile techniques, including handwashing methods, gowning, handling sterile instruments, and maintaining sterility during procedures. Clear guidelines and institutional procedures are recommended to be established to ensure compliance and consistency.

Beyond the radiology department, collaboration with institutional medical safety management units and role-assignment committees is crucial to develop appropriate systems and evaluate task safety.

In IR, which often involves emergency treatments during nights and holidays, additional challenges include ensuring sufficient staffing for expanded roles, revising shift schedules to address emergency needs, and standardizing radiological technologists' technical skills and capabilities.

These new responsibilities should not compromise the quality or safety of existing tasks such as equipment management, imaging operations, reference image provision, and radiation safety management. Institutions are recommended to carefully balance workload expansion with maintaining high standards of care.

### STAT imaging and reporting system

#### *Definition and purpose*

The term "STAT" refers to the urgent interpretation of radiological studies, derived from the Latin word “statim,” meaning "immediately." According to Japanese guidelines, STAT imaging is defined as imaging studies containing findings indicative of conditions with a high risk of mortality if left untreated.

The primary purpose of STAT imaging is to establish a system under the guidance of radiologists that enables radiological technologists to promptly report critical findings. This ensures timely patient care and reduces the risk of mortality due to delayed treatment. As stipulated in the official notice [4], while radiological technologists can report findings, any judgment or diagnosis based on these findings must be made by a physician.

#### *Key principles in STAT imaging guidelines*

These guidelines are intended for hospitals where radiologists are permanently available. Secondly, radiological technologists are tasked with identifying and reporting “image findings.” Diagnoses should be made exclusively by radiologists or other physicians. This approach ensures that the findings reported by technologists do not lead to independent or premature conclusions, maintaining a clear division of roles. During daytime operations, radiological technologists are expected to promptly communicate critical findings to radiologists for confirmation and final diagnosis. In the absence of night-time radiological interpretation systems, the findings reported by technologists should be reviewed by a radiologist no later than the following morning.

**Fig. 1:**
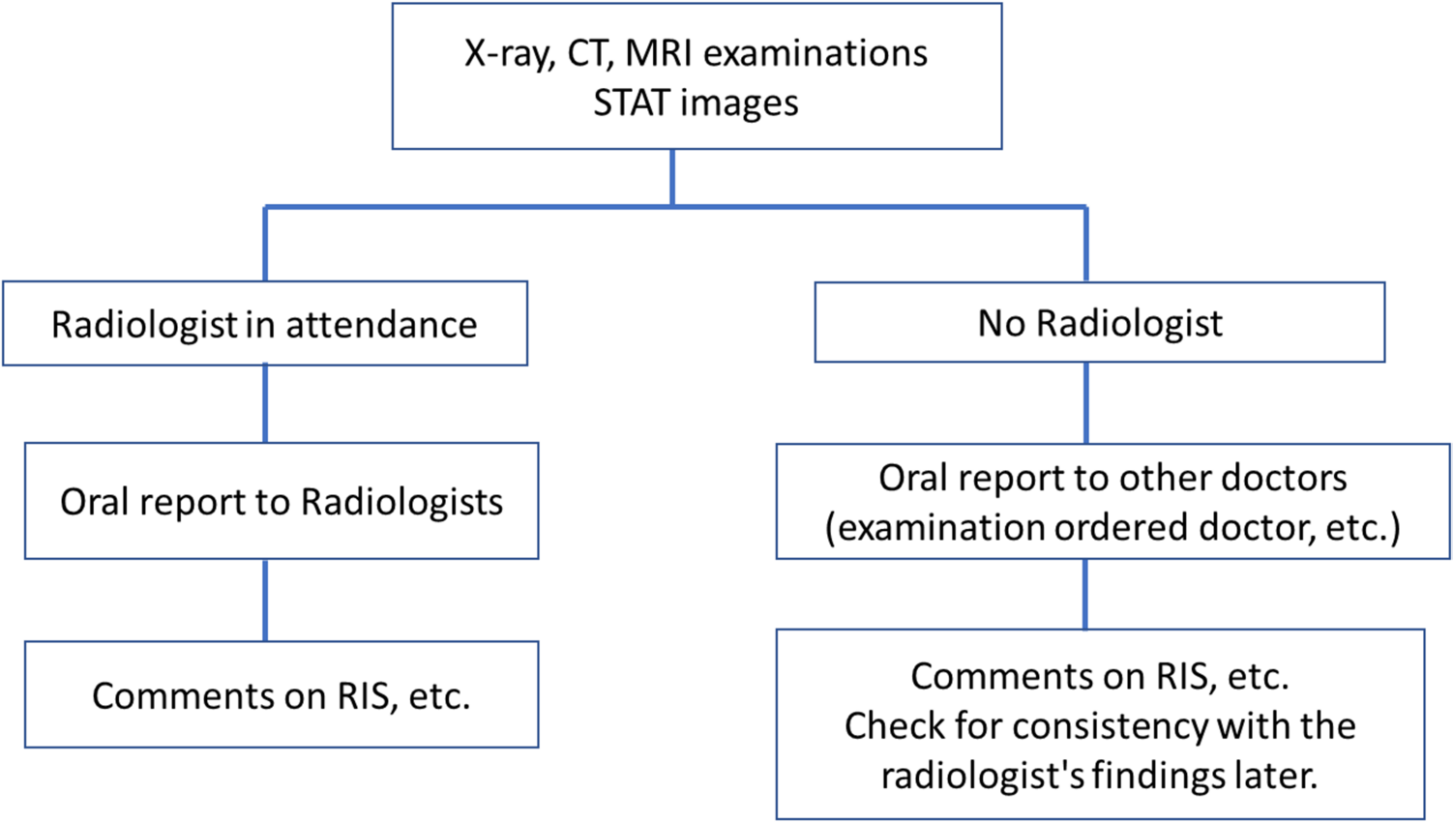
STAT image reporting system by Radiological Technologists. A flow chart indicates the response to STAT imaging cases, distinguishing between scenarios when radiologists are on site and when they are off site. In all cases, radiological technologists document their comments on the RIS, but do not record them in the patient chart

The guidelines cover X-ray, CT, and MRI and outline 13 specific findings that fall under the STAT imaging category (Table 1). The framework for STAT imaging reporting varies by institution but is based on the following recommendations (Fig. 1): The STAT imaging reporting system prioritizes verbal communication as the primary method, based on a consensus established within the facility. Additionally, radiological technologists document their findings in the Radiology Information System (RIS) or the comment section of the reporting system. However, documentation in the report text or medical records is performed by physicians. Subsequently, radiological technologists verify the consistency between their own findings and the interpretations provided by radiologists.

**Table 1:** A list of STAT image findings

Modality	Location	Image Findings	Possible disease
X-ray	Chest	A pneumothorax with intercostal space widening and mediastinal shift toward the contralateral side.	tension pneumothorax
		Intraperitoneal free air (on chest X-ray).	Findings suggestive of gastrointestinal perforation.
	Abdomen	Intraperitoneal free air (on abdominal X-ray).	Findings suggestive of gastrointestinal perforation.
CT	Brain	Intracranial hemorrhage	Intracerebral hemorrhage, subarachnoid hemorrhage, subdural hematoma, and epidural hematoma.
		Brain tumor	Brain tumor
	Chest	A pneumothorax with intercostal space widening and mediastinal shift toward the contralateral side.	Tension pneumothorax
	Abdomen	Intraperitoneal free air	Findings suggestive of gastrointestinal perforation.
		Formation of air-fluid levels and bowel dilatation.	Findings suggestive of bowel obstruction/ileus.
		Intraabdominal hemorrhage	Findings suggestive of intraperitoneal hemorrhage or hematoma formation associated with liver cancer rupture, visceral artery aneurysm rupture, ectopic pregnancy, or blunt abdominal trauma.
	Vascular	An ascending aorta with a diameter of 6 cm or more, a descending aorta with a diameter of 7 cm or more, or an abdominal aorta with a diameter of 5.5 cm or more.	An aortic aneurysm with a high risk of rupture.
MRI	Brain	abnormal high signal intensity on DWI	Cerebral infarction, encephalitis/encephalopathy, demyelinating diseases , etc.
		abnormal signal intensity at extra crania	subarachnoid hemorrhage, subdural hematoma, and epidural hematoma, etc.

The Japan Radiological Society and the Japan Association of Radiological Technologists have collaborated to develop a learning system for technologists. This system uses a radiologist-supervised database (STAT Imaging Reference System) for training. Institutions are encouraged to hold regular conferences on STAT imaging and provide technologists with systematic feedback on their reports. By fostering collaboration between radiologists and technologists and building robust education and feedback systems, institutions can enhance the safety and accuracy of STAT imaging workflows.

### Implementation and effectiveness evaluation of guidelines

To effectively implement task shifting guidelines, a long-term approach is required, including awareness-raising initiatives, the establishment of training systems, the identification of issues, and the development of appropriate countermeasures. To achieve these objectives, it is essential for MHLW, academic societies, educational institutions, and hospitals to collaborate through discussions while fulfilling their respective roles and responsibilities.

To evaluate the effectiveness of task shifting from radiologists to radiological technologists, it is essential to conduct a multifaceted analysis considering aspects such as quality and safety of medical care, work efficiency, education, and patient satisfaction. Specifically, the assessment should include changes in the workload and operational efficiency of radiologists, as well as the overall impact on workflow optimization and time reduction. Additionally, it is necessary to examine whether the workload of radiologic technologists, as the recipients of the shifted tasks, does not increase excessively and whether the burden is appropriately managed. From the perspective of medical quality and safety, it is crucial to determine whether there is an increase in the frequency of incidents or accidents. Evaluation methods should include comparative studies analyzing diagnostic accuracy, work efficiency, and costs before and after task shifting. Furthermore, conducting surveys targeting physicians, technologists, and patients to analyze changes in satisfaction levels serves as an important evaluation measure.

## Conclusion

As one of the pillars of the MHLW's work style reform, the law was amended to allow multiple expansions of work tasks from radiologists to radiological technologists. The extent to which these expanded tasks are selected is left to the discretion of each facility, and the decision is likely to differ depending on various circumstances, such as the number of staff and the placement of equipment within each facility (Table 2). If the radiological technologists who receive the tasks are unable to receive sufficient education and training, the risk to patients increases and they will be unable to carry out examinations and treatments. In each facility, in cooperation with the medical safety department, staff in the radiology department are recommended to discuss and establish an education system, and then examinations and treatments that are safe for patients should be carried out. While implementing task shifting safely, it is essential to simultaneously continue efforts to address the root cause necessitating this approach—the shortage of radiologists.

**Table 2:** Steps for Implementing Task Shifting

Step 1: Analyze Current Workflows
Step 2: Identify Tasks That Can Be Delegated
Step 3: Provide Education and Training from Radiologists to Radiological Technologists
Step 4: Determine Operational Methods Within the Radiology Department
Step 5: Establish In-Hospital Rules in Collaboration with the Hospital’s Medical Safety Department
Step 6: Conduct Trials and Gather Feedback
Step 7: Full Implementation and Continuous Improvement

## References

[1] Nishie A, Kakihara D, Nojo T, Nakamura K, Kuribayashi S, Kadoya M, Ohtomo K, Sugimura K, Honda H. Current radiologist workload and the shortages in Japan: how many full-time radiologists are required? Jpn J Radiol. 2015;33(5):266–72. 25787900 10.1007/s11604-015-0413-6

[2] Kumamaru KK, Machitori A, Koba R, Ijichi S, Nakajima Y, Aoki S. Global and Japanese regional variations in radiologist potential workload for computed tomography and magnetic resonance imaging examinations. Jpn J Radiol. 2018;36(4):273–81. 29453512 10.1007/s11604-018-0724-5

[3] Medical Affairs No. 0528–1, Director-General of the Health Policy Bureau, Ministry of Health, Labour and Welfare, Regarding the promulgation of the "Act to Partially Amend the Medical Care Act and Other Related Laws to Promote the Establishment of Systems for the Efficient Provision of High-Quality and Appropriate Medical Care, https://www.mhlw.go.jp/content/10800000/001256560.pdf

[4] Medical Affairs No. 0930–16, Director-General of the Health Policy Bureau, Ministry of Health, Labour and Welfare,Regarding the Promotion of Task Shifting and Task Sharing Within the Scope Feasible Under the Current System, https://www.hospital.or.jp/pdf/15_20210930_01.pdf

[5] Medical Affairs No. 0430–1, Director-General of the Health Policy Bureau, Ministry of Health, Labour and Welfare, Regarding the Promotion of Team-Based Medical Care Through Collaboration and Coordination Among Healthcare Staff, https://www.mhlw.go.jp/shingi/2010/05/dl/s0512-6h.pdf

[6] Discussion Summary, Council on Promoting Task Shifting and Task Sharing to Advance Work Style Reform for Physicians, December 23, 2020, https://www.mhlw.go.jp/stf/newpage_15678.html

[7] Medical Affairs No. 0709–7, Director-General of the Health Policy Bureau, Ministry of Health, Labour and Welfare, Regarding the Promulgation of the Cabinet Order for Partial Amendment of the Enforcement Order of the Act on Clinical Laboratory Technologists, etc. https://iryou-kinmukankyou.mhlw.go.jp/files/Attachment/222/臨床検査技師等に関する法律施行令の一部を改正する政令等の公布について (医政発0709第7号) .pdf

[8] Yamashiro T, Kumamaru KK, Kido A, Namoto Matsubayashi R, Ota H, Ida M, Aoki S. Joint Committee for Diversity P, Work-Style Reform of the Japan Radiological S, the Japanese College of R: Work-style reform and use of information and communication technology among diagnostic radiologists in Japan: results of the 2018 JRS/JCR joint survey. Jpn J Radiol. 2020;38(7):636–42. 32185671 10.1007/s11604-020-00941-5

[9] Guidelines for Clinical application of Image-Guided Radiation Therapy (IGRT) 2019. https://www.jastro.or.jp/medicalpersonnel/guideline/igrt2019.pdf

